# Visualization and Quantitative 3D Analysis of Intraocular Melanoma and Its Vascularization in a Hamster Eye

**DOI:** 10.3390/ijms19020332

**Published:** 2018-01-24

**Authors:** Bartosz Leszczyński, Martyna Śniegocka, Andrzej Wróbel, Roman Pędrys, Małgorzata Szczygieł, Bożena Romanowska-Dixon, Krystyna Urbańska, Martyna Elas

**Affiliations:** 1 Marian Smoluchowski Institute of Physics, Jagiellonian University, Prof. Stanisława Łojasiewicza 11 Street, 30-348 Krakow, Poland; bartosz.leszczynski@uj.edu.pl (B.L.); andrzej.wrobel@uj.edu.pl (A.W.); roman.pedrys@uj.edu.pl (R.P.); 2 Faculty of Biochemistry, Biophysics and Biotechnology, Jagiellonian University, Gronostajowa 7 Street, 30-387 Krakow, Poland; martyna.sniegocka@doctoral.uj.edu.pl (M.Ś.); gosia.szczygiel@uj.edu.pl (M.S.); krystyna.urbanska@uj.edu.pl (K.U.); 3 Ophthalmology and Ocular Oncology Clinic, University Hospital, Kopernika 38 Street, 31-501 Krakow, Poland; bozena.romanowska-dixon@uj.edu.pl

**Keywords:** imaging, tumor vasculature, ocular tumors, melanoma, hamster, micro-CT, ultrasound

## Abstract

A tumor vasculature network undergoes intense growth and rebuilding during tumor growth. Traditionally, vascular networks are histologically examined using parameters such as vessel density determined from two-dimensional slices of the tumor. Two-dimensional probing of a complicated three-dimensional (3D) structure only provides partial information. Therefore, we propose the use of microcomputed tomography (micro-CT) imaging to analyze the evolution of a tumor vasculature in an experimental ocular tumor model. A Bomirski Hamster Melanoma was implanted in the anterior chamber of a hamster eye. Ultrasound (US) imaging of the same tumor was performed in vivo, and the vascular results obtained using the two methods were compared. Normal ocular tissues, a tumor, and a tumor vascular structure were revealed with high accuracy using micro-CT. The vessels that grew within the tumor were chaotic, leaky, and contained many convoluted micro-vessels and embolizations. They comprised 20–38% of the tumor mass. The blood flow in the larger functional vessels was in the range from 10 to 25 mm/s, as determined by in vivo Doppler US. The micro-CT imaging of the hamster eyeball enabled both qualitative and quantitative 3D analyses of the globe at a histological level. Although the presented images were obtained ex vivo, micro-CT noninvasive imaging is being developed intensively, and high-resolution in vivo imaging is feasible.

## 1. Introduction

Experimental models are essential for evaluating novel diagnostic and therapeutic modalities for prevention, early detection, and/or treatment of cancer. Several animal models of uveal melanoma have been developed and have played a role in advancing our understanding of the disease [1]. An interesting model of ocular melanoma is the bomirski hamster melanoma (BHM), implanted in the anterior chamber in the eye of a Syrian hamster [2,3,4]. Even though it is cutaneous in origin, this allotransplant yields a tumor in up to 96% of cases after implantation in the eye. A significant advantage of this model is that the tumors remain melanotic in vivo and generate pigmented metastases in the lungs.

Tumors require a blood supply, not only for their sustainable growth, but also for hematogenous dissemination [5]. Cancer cells can enter the blood system indirectly through lymphatics, or directly by infiltration of the pathological tumor vessels [6]. Many factors exist that can lead to poor prognosis in uveal melanoma patients, but among the most important ones are genomic abnormalities, aggressive periodic acid-Schiff (PAS) patterns, tumor localization, high microvascular density, and angiogenic activity [7]. One of the reasons why uveal melanomas are very invasive is their ability to create channels that are similar to blood vessels, but that are formed without recruitment of endothelial cells. This phenomenon is called vasculogenic mimicry [5]. Because of the growing interest in antiangiogenic therapeutic agents, learning how to monitor in time the development or regression of tumors in a blood system with as high resolution as possible is crucial. The analysis of spatial distribution of neovessels and mature vessels in human uveal melanoma could improve the efficiency of antiangiogenic therapies [7].

To obtain a proper amount of nutrients for fast tumor development as well as for its dissemination to distant organs, tumors typically trigger an “angiogenic switch” [8]. During angiogenesis in a healthy tissue (for example, in wound healing), a balance in the pro- and anti-angiogenic factors exists that leads to the production of well-organized, regular, and branched ordered system. The tumor microenvironment governs the angiogenesis by overexpression of cytokines and growth factors that cause dysregulation of proper vessel formation [9]. The consequence of very fast remodeling of a tumor vasculature is dilated, tortuous, and leaky vessels with irregular blood flow [10]. Anatomically, tumor microvessels contain regions of increased and reduced vessel density, and much vascular shunting and many haphazard patterns of vessel interconnections are present. The structural abnormalities of tumor vessels are very heterogeneous, and are constantly rearranged with the tumor growth or as a response to therapy.

The typically used histological techniques of microvessel detection are not very effective or easy to apply when it comes to uveal melanomas, because of high tumor pigmentation [5]. Microcomputed tomography (micro-CT) is a well-established method in the field of ex vivo preclinical imaging of highly mineralized structures such as bones or teeth [11,12]. In the case of a soft tissue, the micro-CT imaging quality is limited because of weak X-ray absorption in nonmineralized tissues. To improve the image contrast, various staining agents, which are similar to those commonly used in histology, are applied in micro-CT investigations of soft tissues [13].

The objective of the present study is to evaluate a new approach using micro-CT to investigate the evolution of tumor vasculature during a BHM tumor growth in a hamster eye. We focus on the structure and function of the tumor vessels and their point of origin. Understanding these issues may explain the mechanisms of metastatic spread of these tumors. The mechanism of spreading of an experimental melanoma may be relevant to the mechanisms existing in human ocular tumors, especially potential blood-vessel invasion in uveal melanoma. The spatial analysis of a tumor vasculature may reveal the tumor-vessel development, morphology of the vascular network, and vessel distribution within the tumor as well as the determination of irregularities in the vascular network.

## 2. Results

**Figure 1:**
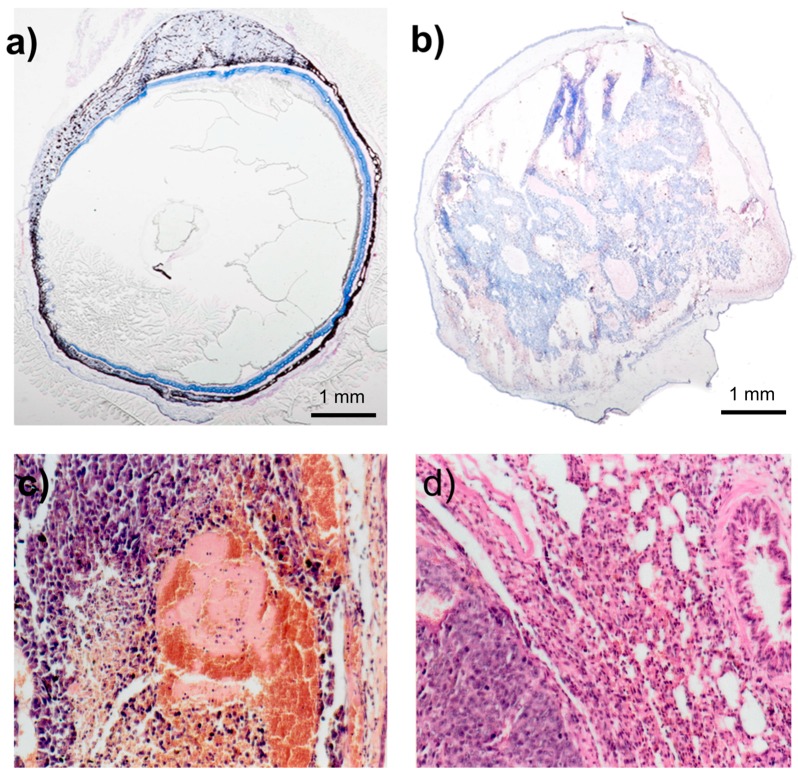
H&E-stained cross section through the hamster eye with BHM implanted into the anterior chamber. (**a**) Tumor grows in the anterior chamber only, and the choroid is clearly visible. Enucleated on day 9; (**b**) tumor masses fill up the whole eyeball, destroying the normal structures. The diameter of the eyeball is approximately 20% bigger. Enucleated on day 13; (**c**) H&E-stained tumor within the anterior chamber, magnification 360×; (**d**) H&E staining of the lung metastasis, magnification 360×.

The BHM tumor quickly invades the eyeball. The BHM tumor implanted into the anterior chamber of the hamster eye very quickly grows [4]. Within 5–7 days, the whole anterior chamber is filled out (Figure 1a, day 6 after implantation) with a normal choroid clearly visible. Gradually, the whole eyeball fills with tumor masses (Figure 1b, day 11 after implantation), which deform the eyeball, and the normal ocular structures, e.g., the lens and vitreous body, are no longer discernible. Some necrotic areas are seen within the tumor mass (Figure 1c). Spontaneous metastases, spreading mainly to the lung, remain pigmented and resemble the primary tumor (Figure 1d).

**Figure 2:**
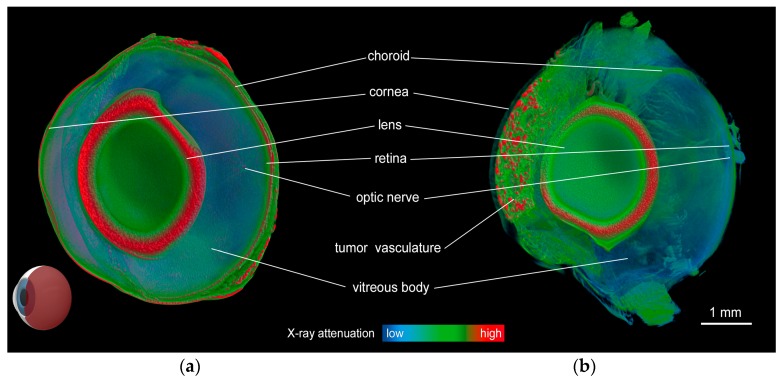
Micro-CT imaging. Volume-rendered 3D image of the hamster eyeball without melanoma (**a**); and with a medium-sized melanoma growing in the anterior chamber stained in Lugol solution for 96 h (**b**). The models are virtually cut in the sagittal plane, as shown in the model eyeball in the lower left corner. Pathological vascularization and tumor masses are clearly visible.

The micro-CT results are shown in Figure 2. The volume rendering of a 3D model of the normal eye (Figure 2a) shows very efficient contrast enhancement because of the applied staining procedure. Using a combination of staining and virtual coloration (by modifying the RGB transfer functions of the 3D model), all fine details of the hamster eye anatomy are clearly visible, as shown in Figure 2. The medium-sized tumor shown in Figure 2b exhibits a clearly defined vasculature network within the tumor masses localized in the anterior chamber of the eye. A representative cut through a 3D image is shown in Figure 2a,b.

**Figure 3:**
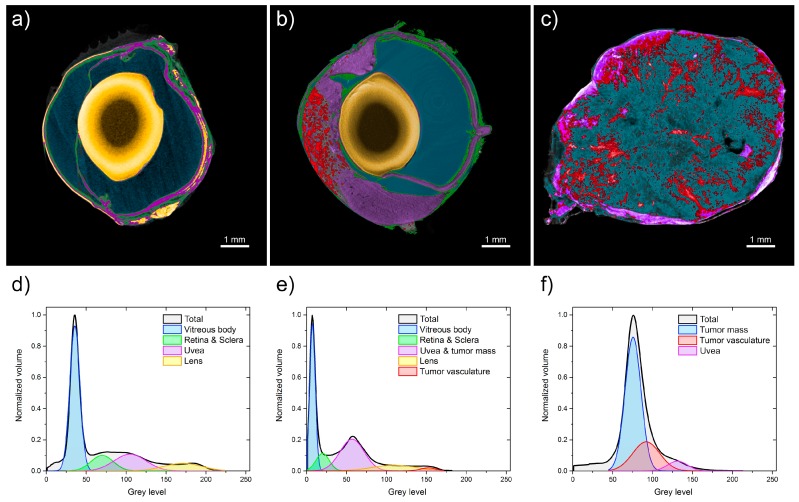
Analysis of micro-CT images. Volume-rendered 3D image of (**a**) a normal hamster eyeball and with melanoma at different stages, (**b**) medium-sized tumor, and (**c**) large tumor. Gray-value histograms based on the micro-CT images of a hamster eyeball stained in Lugol solution for 96 h: (**d**) control group, and (**e**) eyeball with medium melanoma and (**f**) large melanoma. The fitted Gauss curves correspond to the main anatomical parts of the samples, i.e., vitreous body, retina, and sclera; choroid, lens, tumor vasculature, and tumor mass.

Three micro-CT images of the normal eyeball, medium-sized melanoma, and large melanoma are compared and shown in Figure 3. Figure 3c shows that the volume of the eye with the tumor is overdominated by a pathological vascularization net and a widespread tumor mass. All ocular structures are invaded by the melanoma, and the lens is not visible in Figure 3c. The vascular network revealed by micro-CT imaging is very dense in both the medium-sized and large tumors, particularly in the outer layers. The vasculature reaches up to 38% of the tumor volume. In the large tumor, both heavily vascularized and some avascular areas are seen (Figure 3b,c). Morphologically, irregular structures in the vascular network are observed, with highly varying diameters and lengths of vessels, with many local constrictions and dilatations, and with no visible hierarchy. The range of the tumor-vessel diameter was between 10 and 120 µm, although the upper limit might be an artifact of the algorithm, by counting two close vessels as one. The separation between the vessels was even more irregular. These parameter values spread out from 10 to 700 µm. However, a majority of the vessel-separation values cumulate between 100 and 300 µm (not shown).

The gray-level histograms shown in Figure 3d–f characterize the whole volume of each sample. A visible difference is clearly seen among the three histograms. The nonparametric Mann–Whitney test proves significant statistical differences among these histograms at *p* = 0.005. Three Gauss functions are fitted to the histograms. The fitted-curve parameters are listed in Table 1. Each fitted curve corresponds to a particular part of the sample.

Figure 3d shows a very wide distribution of the gray level in the control-group sample extending from 20–200 gray-level indices. We can easily distinguish three separable sections as follows: vitreous body, broadly understood ocular tissues, and lens capsule. Analysis of the eyeball with a medium-sized tumor reveals a histogram from 0 to 170 with a smaller vitreous body and larger choroid and tumor mass compared with a normal eye (Figure 3e). Figure 3f shows the gray-level distribution in the eye sample with a large melanoma. In contrast to Figure 3d, the overall shape of the histogram in this case is completely different. It extends from 50–150 gray-level indices, and no normal anatomical structure can be detected. The tumor vasculature is estimated at 2.5% of the total globe volume in the medium-size-tumor eyeball and at 25% in the large-tumor eyeball. Vessel density is 35–46 per mm^2^ in medium-sized tumors, whereas in large tumors it decreases to 13–17 per mm^2^ (Table 2). No lens is visible within the eyeball with a large tumor, which is probably an artifact of the eyeball preparation.

**Figure 4:**
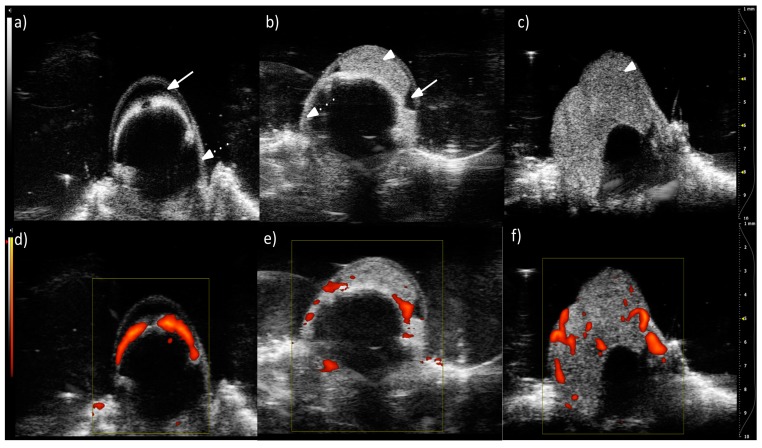
Single cross-section from 3D US images of the tumor in the eye of a living hamster. B-mode images showing a control eyeball (**a**); eyeball with a medium-sized tumor (**b**); and large tumor (**c**). Visible structures of the eye are indicated: anterior chamber (solid arrow), choroid (dotted arrow), tumor mass (arrowhead). In the lower panel, the Doppler mode is presented, showing vascular blood flow (color) in a control eyeball (**d**); eyeball with a medium-sized tumor (**e**); and large tumor (**f**). All colored areas correspond to blood flow. Darker colors indicate lower frequency signals. Scale on the right is in mm.

Prior to micro-CT, ultrasonographic imaging was performed in living animals to monitor tumor growth and vasculature development. The US B-mode live imaging of the same hamster eyeballs as shown in Figure 3 reveals the location of the eye structures (anterior chamber, sclera, and choroid) and the tumor [Figure 4a–c]. In the Doppler mode, we observe a choroid with a strong blood flow in the normal eye. In the tumors, numerous vessels with blood flow are seen [Figure 4e,f]. These vessels have irregular positions, shapes, and sizes. Tumor vessel density determined from US images was 9–21 per mm^2^, whereas in large tumors it was 2–10 per mm^2^ (Table 2). The lower values were expected, as the resolution is much lower in the US than in the micro-CT. Tumor vessel volume expressed as a percentage of tumor volume decreased from 30% to 19% as determined by US. Most authors imply that this parameter should be stable [14] or increase [15] with tumor growth. However, in other experimental models, we have also noticed a gradual diminishing of the vasculature in relation to tumor volume, due to fast tumor growth and the development of necrosis. Tumor vessel volume calculated from micro-CT, however, was very low for a medium-sized tumor, at 6.4%. Such a low value resulted from the presence of large avascular regions, localized outside the anterior chamber (Figure 3b). Tumor vessel volume calculated as a percentage of the anterior chamber volume, the primary site of this tumor, was 14.8%. In contrast, a very high value of 30% from US for a medium size tumor resulted from the inability to discern between tumor and normal vessels, surrounding the tumor, originating from uvea, as well as from a lower spatial resolution of the method.

**Figure 5:**
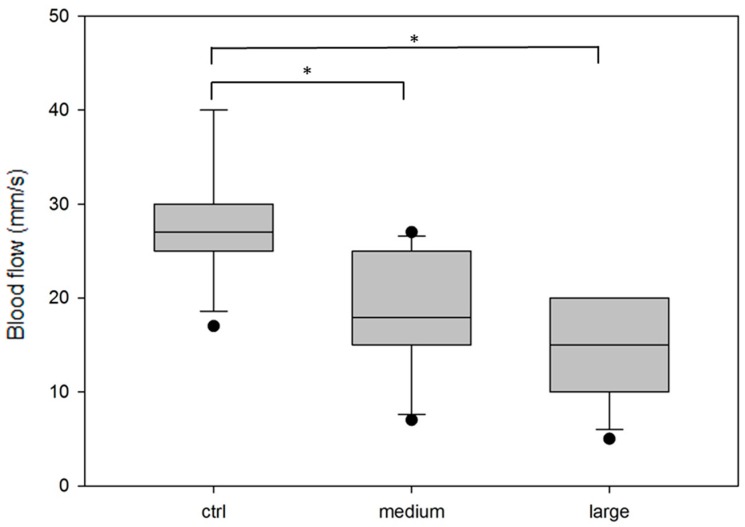
Range of the blood flow in the vasculature measured from Doppler USG images in the eyeballs without a tumor (ctrl) or in the tumor vasculature in medium-size and large tumors. The blood flow is measured in approximately 10 vessels from each experimental group. The box range is between 25th and 75th percentile. The line within the box indicates the median, the error bars indicate the 10th and 90th percentile, and the black dots indicate the outlying points. The statistical significance of the differences between the mean values is tested using one-way analysis of variance (Holm–Sidak test). Asterisks stand for *p* < 0.05.

The blood flow of 10 randomly chosen vessels from the control and each tumor group was measured. Figure 5 shows that the tumor blood flow was lower than the blood flow in a normal choroid (mean value in the control is 28.3 mm/s versus 17.9 mm/s in the medium tumor and 14 mm/s in the large tumor). The statistical significance of the differences between the mean values was tested using one-way analysis of variance (Holm–Sidak test). A representative slice from the 3D images is shown in Figure 4.

## 3. Discussion

The ability to monitor the changes in the tumor blood-vessel development is essential to controlling the effectiveness of anticancer therapies [16]. In this paper, we proposed two different methods of blood-vessel visualization of tumors that grow in the anterior chamber of a hamster eye. Other models of ocular melanoma are available, such as human primary uveal melanoma cell lines Mel290, Mel270, or OCM8; mouse melanoma B16LS9; and Queens or B16F10 cells, which can be implanted into the ciliary body, choroid, or vitreous cavity in a murine eyeball [17,18]. The main advantage of our model is that the hamster eyeball is approximately 2.5 times bigger than that of a mouse. Therefore, we can observe the development of vascularization in tumors with different sizes, which is very important because it provides additional information, such as the size of the tumor when development of new vessels is induced [17] or the influence of hypoxia in large tumors on the effectiveness of the therapy [19].

**Figure 6:**
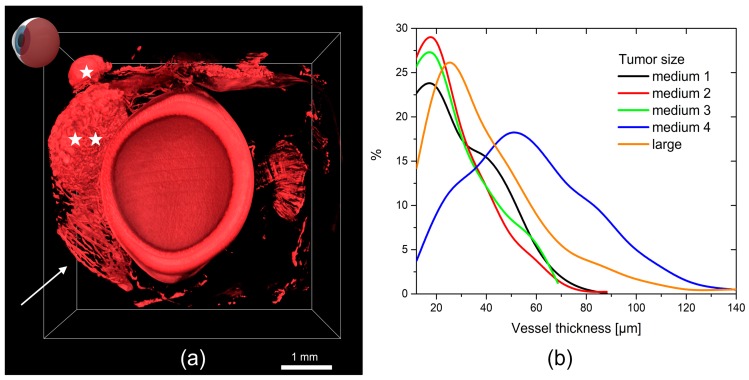
(**a**) micro-CT image of the vascular structures of a medium-sized BHM melanoma with lens. (★) Extraocular extension of an intraocular melanoma. (★★) Medium-sized tumor vascularization. The arrow points to the structure of a normal iris vasculature; (**b**) distribution of the vessel size in the eyeballs with BHM tumors.

Micro-CT, together with iodine staining, provides excellent results for investigation of BHM in a hamster eye. This is the only method that enables both qualitative and quantitative analyses of the globe at a histological level in 3D [20]. Visualization of tumor vascularization against the background of a normal globe anatomy provides a unique opportunity to study the spatial relationship of BHM. In Figure 6a, only tumor vasculature is shown (the same tumor as in Figure 3b), revealing a convoluted network of tumor vessels, as well as some remaining normal vasculature. According to micro-CT images, a wide range of morphometric parameters can be calculated [21]. In particular, vessel-diameter distribution and separation provide information on the stage of the neoplastic process. Vessel separation in most studied tumors was between 10 and 90 µm, with an exception of a single tumor with higher values, probably due to overstaining and difficulty in separating vessels (Figure 6b). The 3D analysis ability and isotropic resolution at the micrometer level result in a high statistical accuracy for the calculated parameters.

We need to mention that staining microtomography is specially designed for ex vivo samples [22]. However, micro-CT is a nondestructive method, which means that the globe can be examined by other methods at a later time.

Micro-CT investigation of the vascular network produced by ocular tumor is a novel approach for this method. Only a few papers have been published in which micro-CT is employed for globe visualization [23].

Ultrasonography has become an essential technique for diagnosis of many different pathologies in all fields of medicine, including ophthalmology [24]. Its widespread use is due to its noninvasiveness, relatively easy management, fast results, and satisfactory ratio of costs and effects [25]. The power Doppler mode provides the possibility to not only see the blood vessels, but also measure the blood velocity. High-frequency Doppler imaging offers resolution that is sufficiently good for observing the morphology and flow in vessels as small as 15 µm [26]. This is important information in terms of cancer biology, therapy effectiveness, and survival prognosis, because vascular density in some human cancers as observed by Doppler US is often correlated with metastatic potential. Although in vivo visualization and quantification of vessels can be performed using many different methods [27] (including positron emission tomography, single-photon emission computed tomography, or magnetic resonance imaging), power Doppler US appears to be the quickest and cheapest in vivo assessment of angiogenesis in human or animal tumors. To the best of our knowledge, only one case of US imaging of intraocular melanoma in a hamster has been described and published [28]. Interestingly, it was also a melanoma of cutaneous origin, which is similar to the BHM case. Administration of US contrast agents (such as microbubbles) makes possible the detection of vessels with higher contrast, compared with measurements without an enhancing agent [29]. In the present study, which does not use microbubble contrast, we observed vessels of approximately 70 µm both in the normal choroid and in the tumor tissue.

We compared the tumor growth in the eyeball at different stages of the tumor growth. A disorganized and very irregular pattern with no vessel hierarchy and a very heterogeneous vessel size was observed. This result is in contrast to the normal hamster-eye vascular corrosion cast study, which showed a very clear hierarchy of the vasculature with vessel-size range from 3.6 to 50 µm [30].

The tumor vasculature is characterized by tortuous courses in the blood vessels, especially venules and small veins. Uneven shapes and variable diameters of dilated venous vessels are present. The venules and sinusoidal capillaries, which exhibit heterogeneous intratumor density, are intensively interconnected. The tumor vasculature of the BHM tumor that grows in the eye as revealed by the micro-CT imaging is comparable with the vascular corrosion cast study we performed previously [31]. The corrosion cast requires filling out the vasculature with a polymer resin and, after its solidification, removal of the tissues. Resin, injected with some pressure, may lead to small-vessel disruption and extravasation. In contrast, micro-CT is not an invasive technique, i.e., the eyeball is imaged as a whole after incubation with iodine contrast. In addition to the vasculature, all other structures of the eye are visible. Both techniques yield very similar results in terms of the vasculature structure.

Usually, tumor vasculature, expressed as a percentage of the tumor volume, decreases with tumor growth. This was confirmed by a decrease in the vessel density, as well as tumor vessel to tumor volume ratio, as determined by US. The more precise micro-CT determination revealed a reversed dependence due to large avascular areas in the medium-size tumor.

The blood flow in the vessels in a tumor is a very important parameter, because it helps determine the tumor microenvironment and tumor response to different therapeutic approaches and is inseparably connected with chemotherapeutic-agent delivery [32]. A high individual variation in normal orbital vascular patterns is possible [33]; hence, it would not be surprising if the variation is even higher in pathological structures. Additionally, Yang et al. [34] described that pulsatile ocular blood flow can occur in the presence of melanoma. Nevertheless, we are able to determine a statistically significant difference in the velocity of blood flow in melanoma tumors compared with that in normal eyeballs. The lower velocity of blood flow in the tumors than that in a normal tissue is probably caused by abnormal vessel rearrangements, lack of hierarchy, and variable diameters.

## 4. Materials and Methods

### 4.1. In Vivo Model

BHM tumor is a spontaneously developed cutaneous melanoma [2,3] and has been maintained in our laboratory for many years. 0.5–1 mm fragments of a BHM tumor were implanted into the anterior chamber of a female, 14–16 weeks old hamster eye using a surgical microscope. The growth of the tumor and condition of the animals were observed daily. The volumes of the tumor and vessels were measured daily using the surgical magnifier and Doppler US. The eyeballs were enucleated when the anterior chamber was completely filled with a tumor mass, or when the cornea was significantly destroyed. The amount and localization of metastases were determined during a post-mortem examination. Animal experiments were performed in accordance to EU and national regulations for animal experimentation, Local Committee for Animal Research approval no 90/2014 (21/05/2014), 264/2015 (15/12/2015), and 265/2015 (15/12/2015).

### 4.2. Histology

The eyeballs were put in an optimal cutting-temperature compound (Cryomatrix, Thermo Fisher Scientific, Waltham, MA, USA) and immediately frozen in liquid nitrogen. The tissue was cut into 6-mm-thick slices using a cryostat. The frozen sections were stained with hematoxylin and eosin (H&E) stain in the following manner: fixing in cold ethanol for each concentration (96%, 95%, 80%, and 70%) for 1 min, rinsing in distilled water, staining in hematoxylin for 5 min, rinsing in tap water for 15 min, putting in 95% ethanol for 1 min, staining in eosin for 1.5 min, and repeating the process by selecting the ethanol concentration (95%, 80%, and 70%). The slices were protected using a glass cover. All the chemicals were purchased from POCh, Gliwice, Poland.

### 4.3. Micro-CT Protocol

The micro-CT investigation was performed for three groups of hamster eyeballs. The first group contained the globes with medium-sized tumor (*n* = 3), the second group were globes with an advanced neoplastic process (*n* = 2). The globes without the tumor served as control (*n* = 2). After the enucleation process, the eyeballs of both groups were fixed in 10% formaldehyde. Then, they were washed and stained in 100% Lugol solution at 4 °C for 96 h. To perform microtomographic measurements, the samples were mounted on a specially designed three-dimensional printed holder to prevent artifacts resulting from possible sample motion during scanning. Micro-CT scanning was performed using the SkyScan 1172 instrument (SkyScan, Kontich, Belgium). The overall scanning parameters were set as follows: X-ray energy of 80 keV, rotation step of 0.2°, image pixel size of 7 µm, and averaging of 12 frames for each image projection. Image reconstruction and analysis were performed using the SkyScan software package (Nrecon v. 1.7.1.0 and CTAnalyser v. 1.16.1.0, Bruker microCT, Kontich, Belgium). Statistical analysis was done using OriginPro software (OriginLab, v. 2016, Northampton, MA, USA). The volume rendering models of both eyes were produced using the CTVox software by SkyScan (v. 3.3.0, Bruker microCT, Kontich, Belgium). Vessel density was calculated based on a number of vessels per mm^2^ in 10 randomly selected 2D cross-sections, analogically to the histological determination.

### 4.4. US Protocol and Data Analysis

A high-resolution ultrasound (US) imaging system specially designed for examination of small experimental animals (Vevo 2100, FujiFilm Visual Sonics, Toronto, ON, Canada) was used. An MS-550D (with a center operating frequency of 22–55 MHz and spatial resolution of 20 × 20 × 170 µm) probe was used to acquire all images. For the initial confirmation of a tumor, a B-mode US (grayscale) was performed at a central frequency 40 MHz and a gain of 18 dB. Doppler imaging was used in the US test to detect the presence of blood flow and to evaluate the direction and speed of flow in vessels whose diameter was larger than 30 μm. Color and power Doppler imaging at a central frequency of 32 MHz and pulse repetition frequency of 3–4 kHz was performed. A clear US gel was centrifuged (to remove air bubbles) and used to provide photoacoustic coupling between the probe and tumor. The left eyes of all animals were scanned. We obtained 3D images, each containing 40 scans per eye, at a 0.171 mm step using a steady-arm-held transducer. The initial transducer position was set in the maximal diameter of an eyeball. To perform US imaging, the animals were anesthetized using isofluorane (2–3%). The animals were secured to a heated animal-handling platform, which allows monitoring of the electrocardiogram, respiration, and body temperature.

The tumor morphology (echogenicity, structure, shape, and volume of the tumor), as well as the tumor vascularity (the presence of a power Doppler signal determined by a color frequency map, the circulatory pattern, and the total volume of the vessels in the tumor), were calculated. The region of interest (ROI) in each slice was manually selected. The vessel and tumor volumes were calculated based on the following formula:

V = area ROI × height of slice(1)

The total tumor volume represented the sum of the sliced-tumor volume and is expressed in cubic millimeters. Vessel density was evaluated based on a number of vessels per mm^2^ in 10 randomly selected 2D cross-sections. For the data analysis, home-built Matlab scripts were used.

### 4.5. Statistical Analysis

Imaged-data analysis was performed using commercial software (Vevo) or home-built image analysis software (vasculature data from ultrasonography). The blood-flow data from a single tumor were presented as a range of values. The nonparametric Mann–Whitney test was used to compare the histograms.

## 5. Conclusions

In conclusion, micro-CT and Doppler US are effective methods for visualization of tumor vasculature. The micro-CT visualization allows ex vivo determination of the tumor location, size, and vascularization with high accuracy. Meanwhile, the Doppler US provides the possibility of in vivo measurements of the same features, but with significantly lower resolution. The vessels that grew within a BHM tumor, which originated from the choroid, were chaotic, leaky, and contained many tortuous micro-vessels and embolizations. The vessels in the medium-sized tumors reached up to 30% of the tumor mass, and those in the large tumors reached up to 19%. Many of the larger vessels in the BHM tumors, as observed from the Doppler US, were functional with a blood flow in the range of 10 to 25 mm/s. The speed of the blood flow decreased with the tumor growth. The investigated animal model of the ocular melanoma could be extrapolated to a human uveal melanoma in terms of blood-vessel wall invasion, mechanisms of cancer-cell migration, and angiogenesis process or response to anti-cancer therapy.

## Figures and Tables

**Table 1:** Parameters of the Gauss functions fitted to gray-level histograms of the representative hamster eyes without melanoma, with a medium-sized tumor, and with a large tumor. The results are expressed as the mean values ± Standard Deviation. Relative area was calculated as a ratio of corresponding peak area to total area. * (asterisks) indicate that medium tumor mass includes mass of identically stained uvea. The same eyeballs are shown in Figure 3.

Analyzed Eyeball	Vitreous Body		Retina & Sclera		Uvea		Lens		Tumor Vasculature		Tumor Mass	
	x_c_ ± SD (g.l.)	Relative Area (%)	x_c_ ± SD (g.l.)	Relative Area (%)	x_c_ ± SD (g.l.)	Relative Area (%)	x_c_ ± SD (g.l.)	Relative Area (%)	x_c_ ± SD (g.l.)	Relative Area (%)	x_c_ ± SD (g.l.)	Relative Area (%)
Normal eyeball	36 ± 6	56	70 ± 14	13	105 ± 20	20	171 ± 24	11	-	-	-	-
Medium tumor	7 ± 4	39	20 ± 8	11	*	*	110 ± 28	12	153 ± 10	2	57 ± 15 *	36
Large tumor	-	-	-	-	131 ± 13	6	-	-	92 ± 17	25	76 ± 10	69

**Table 2:** Comparison of tumor vessel density and percentage of tumor vessel volume within the tumor estimated with micro-CT and US. Tumor vessel density is expressed as a range of all values.

Tumor size	Tumor Vessel Density as Determined by		Tumor Vasculature Volume as % of Tumor Volume	
	Micro-CT	US	Micro-CT	US
Medium tumor	35–46/mm^2^	9–21/mm^2^	6.42%	30.21%
Large tumor	13–17/mm^2^	2–10/mm^2^	37.28%	19.24%

## Abbreviations

3D	Three-dimensional
Micro-CT	Microcomputed tomography
US	Ultrasound
BHM	Bomirski Hamster Melanoma
PAS	Periodic acid-Schiff
H&E	Hematoxylin and eosin
ROI	Region of interest
RGB	Red Green Blue
